# Piloting racial bias training for hospital emergency department providers treating patients with opioid use disorder

**DOI:** 10.1093/haschl/qxae049

**Published:** 2024-04-24

**Authors:** Jason B Gibbons, Samantha J Harris, Olivia K Sugarman, Eric G Hulsey, Julie Rwan, Esther M Rosner, Brendan Saloner

**Affiliations:** Department of Health Systems, Management and Policy, Colorado School of Public Health, University of Colorado Anschutz Medical Campus, Aurora, CO 80045, United States; Department of Health Policy and Management, Bloomberg School of Public Health, Johns Hopkins University, Baltimore, MD 21205, United States; Department of Health Policy and Management, Bloomberg School of Public Health, Johns Hopkins University, Baltimore, MD 21205, United States; Overdose Prevention Program, Vital Strategies, New York, NY 10005, United States; Overdose Prevention Program, Vital Strategies, New York, NY 10005, United States; Overdose Prevention Program, Vital Strategies, New York, NY 10005, United States; Department of Health Policy and Management, Bloomberg School of Public Health, Johns Hopkins University, Baltimore, MD 21205, United States

**Keywords:** opioid use disorder, implicit bias, racial bias training, health equity

## Abstract

Racial disparities in opioid overdose have increased in recent years. Several studies have linked these disparities to health care providers’ inequitable delivery of opioid use disorder (OUD) services. In response, health care policymakers and systems have designed new programs to improve equitable OUD care delivery. Racial bias training has been 1 commonly utilized program. Racial bias training educates providers about the existence of racial disparities in the treatment of people who use drugs and the role of implicit bias. Our study evaluates a pilot racial bias training delivered to 25 hospital emergency providers treating patients with OUDs in 2 hospitals in Detroit, Michigan. We conducted a 3-part survey, including a baseline assessment, post-training assessment, and a 2-month follow-up to evaluate the acceptability and feasibility of scaling the racial bias training to larger audiences. We also investigate preliminary data on changes in self-awareness of implicit bias, knowledge of training content, and equity in care delivery to patients with OUD. Using qualitative survey response data, we found that training participants were satisfied with the content and quality of the training and especially valued the small-group discussions, motivational interviewing, and historical context.

## Introduction

Racial bias by US health care providers is a contributor to racial inequity in health and treatment outcomes.^1-9^ Bias can result from many factors, including implicit biases among health care providers and a lack of diversity in the medical field.^4,5^ Black patients with opioid use disorder (OUD) are especially at risk of receiving inequitable treatment due to both racial bias and stigma against people with substance use disorder.^10^ Black people who use drugs have experienced a nearly 9-fold increase in fatal opioid overdose over the last decade, and in 2020, Black overdose deaths exceeded White overdose deaths for the first time in history.^11^ Racial inequity in treatment driven by bias and stigma has been linked to rising racial disparities in opioid overdoses,^12,13^ so developing and evaluating programs to reduce bias and stigma among providers is timely.

Equipping health care providers with the knowledge and self-reflection tools to improve their cultural awareness could be an important step to reducing rising racial opioid overdose disparities. This work is supported by a growing call by US health care organizations, researchers, and policymakers to highlight racial bias in medicine and work to reform clinical practice to improve treatment equity.^7,14^ One widespread practice is implementing and requiring racial bias training programs in health systems and hospitals.^15^ Racial bias training aims to help health care providers understand and overcome their own implicit biases (ie, unconscious perspectives resulting in disparities in the kinds of treatment and treatment intensities delivered to patients) that have been shown to lead to disparities in mortality rates and other outcomes.^16,17^

Several studies have evaluated the effectiveness of racial bias training in medicine in alerting providers to their potentially discriminatory care practices. In a study of racial bias training delivered to National Health Service providers in the United Kingdom, providers receiving the training had higher racial bias awareness and cultural humility after the training.^18^ A separate evaluation of implicit bias training delivered to first-year medical students found that students who received the training exhibited less bias after the training relative to nonparticipants.^19^ A third study found a 9% decrease in implicit bias attitudes after multicultural training was delivered to counseling professionals.^20^ A fourth training evaluation noted that providers reported improved bias awareness, commitment to improving equitable care, and a desire to continue training and monitoring implicit bias.^21^ However, a fifth showed that occupational therapists who received virtual cultural humility training exhibited similar levels of bias before and after training.^22^ Despite some promising findings, more research evaluating implicit bias training interventions is needed to quantify their impact in the context of OUD where biases against people who use drugs and minoritized populations are prevalent.

In 2022, the Community Foundation for Southeast Michigan, a philanthropic organization supporting the region, partnered with New Detroit, a racial justice organization, to develop a comprehensive racial equity training program for health care providers in emergency departments (EDs) treating patients with OUDs.^23^ The training, called Just Care, contained modules on implicit bias, motivational interviewing, inequity in the medical treatment of Black patients, and disparities in OUD outcomes. New Detroit designed the training in collaboration with Global Diversified Awareness Project LLC, which provided expertise on OUD, especially lived experience. The training arrived just after new legislation passed in the state that mandates all professions licensed under the Public Health Code to receive at least 2 hours of implicit bias training every 5 years.^24^

Our study evaluates the New Detroit Just Care racial bias training pilot delivered to health care providers working in hospital EDs in Detroit, Michigan. The evaluation focuses on the feasibility and acceptability of the pilot training and the potential to scale it to larger groups of participants. We additionally assessed preliminary data on training knowledge, perceptions, and care delivery changes.

## Data and methods

### Study design

We designed a 3-part survey to assess provider perspectives on disparities in OUD treatment, patient–provider relationships, knowledge gained from racial bias training, and changes in OUD care delivery. The survey included both multiple-choice questions with Likert-scale agreement options and open-ended questions, where responses were prompted to explain their choice to a multiple-choice question or were asked to speak to their experiences. The survey included 3 primary required components: (1) a baseline assessment survey administered the day of the training just before the participants received the training (ie, minutes before the training began), (2) a post-training survey administered immediately after the training, and (3) a 2-month follow-up survey. Some questions were asked in only 1 or 2 of the surveys, while other questions were used in each of the 3 surveys. The main domains included patient–provider relationships, comfort in talking about race, the lived experience of Black patients with OUD, disparities in interactions with patients being treated for OUD, and disparities in the kinds of OUD treatments and intensities being delivered. Questions were adapted from validated instruments in other assessments of racial bias.^18,19,25,26^ We also created new questions specific to topics covered during the racial bias training and in the context of Michigan. The complete set of 53 survey questions and their inclusion in each training component is provided in Appendix Table S1. The Community Foundation for Southeast Michigan paid participants $1200 each for participation in the training and completion of each survey component. The payment was delivered after all 3 surveys had been completed.

After participants completed the surveys, we conducted a univariate analysis to evaluate provider responses within each component. Free responses were compiled and reviewed for thematic trends. We then compared changes in survey responses for questions included across each component. This allowed us to determine how provider responses evolved.

### Study population

Our study participant population included 25 health care providers delivering services in 2 hospital EDs in southeast Michigan. Southeast Michigan has the highest proportion of Black residents in the state; in Detroit alone, Black people who use drugs accounted for 72% of ED opioid overdose encounters and more than 75% of fatal opioid overdoses in 2019.^27^ The Black population in Detroit represents 77.8% of the full population according to 2022 US Census data.^28^

Participants were mostly White (88%), non-Hispanic (96%), male (56%), and middle-aged (ie, average age of 39.6 years). Participant specialties were mixed between ED physicians (64%) and physician assistants (36%) Of these participants, 22 (88%) were buprenorphine waivered, 18 (72%) had previously prescribed buprenorphine in the ED, 17 (68%) had inducted patients buprenorphine in the ED, and 23 (92%) had previously treated a patient experiencing an overdose. Moreover, 15 (60%) had previously taken racial bias training, while 7 (28%) had taken training specific to addiction stigma (see Appendix Table S2).

### Data analysis

We conducted a univariate analysis to summarize responses to the multiple-choice survey questions. We obtained average values for each survey response within each item to determine the distribution of answers to individual questions. For questions using Likert scales, we aggregated responses to look at changes in agreement with each item over time. For questions adapted from a study on implicit bias and negative attitudes toward patients with sickle cell disease,^25^ we started by comparing the proportion of participants that said more than 50% of their Black vs White patients fell into a specific category (ie, satisfying to take care of) in the post-training and follow-up training surveys, separately. We then compared differences in those differences to evaluate changes over time. In our Results section, we report on all changes in perceptions between the survey components, even if they were not statistically significant. For analyses looking at changes in the proportion of respondents in agreement with certain survey questions, each additional participant in agreement reflected a 4-percentage-point change (ie, 1/25 = 4%). To test the statistical significance of changes in responses over time, we used McNemar's test for paired nominal data.

The survey instruments also included several free-text response questions; participants were asked to explain their selection for a multiple-choice question throughout the survey or asked an open-ended question. Responses to open-ended survey questions were analyzed for common themes. Exemplary quotes on the related topic are reported below. Additional quotes and data points are provided in Appendix Tables S3–S6 and Appendix Figures S1 and S2.

## Results

### Feasibility and acceptability of the pilot evaluation

All 25 recruited participants to attend the training were present throughout the entire duration of the training and completed all required questions across each of the 3 surveys. For open-response questions eliciting additional clarification of multiple-choice responses, at least 60% of participants provided additional written context across each of the surveys.

In the post-training survey, a total of 22 participants (88%) responded that they found the training useful in communicating their perceptions of race, and 25 (100%) in their understanding of other people's perceptions of race (see Appendix Figure S1). Some exemplary quotes are provided below (see Appendix Table S4):

“I felt that the open dialogue felt like a safe place to discuss individual perceptions regarding race and the conversation was very helpful in pointing things out that I may not have otherwise considered or thought were a big deal.” (Participant 21)

“It was important to see how other providers with privilege experience working with minority populations.” (Participant 15)

Participants also predominantly agreed that the “no-fault environment was useful to reflect about the impact of race” (23/25 strongly agree and 2/25 agree), that they “felt that a safe space was created to have this conversation on race” (21/25 strongly agree, 3/25 agree, 1/25 neither agree nor disagree), and that “this session increased my level of comfort to have a conversation about race using this type of small-group format (18/25 strongly agree, 5/25 agree, 2/25 neither agree nor disagree). There was also majority agreement that “the group dialogue today helped to promote racial healing” (15 strongly agree, 6 agree, and 4 neither agree nor disagree) and that “this session increased my awareness of implicit or unconscious bias” (16 strongly agree, 6 agree, and 3 neither agree nor disagree) (see Appendix Figure S2).

With regard to perceptions on the most useful parts of the training, most participants responded that the small-group discussions and interactive dialogue components of the training (11/20 participants) helped them learn and engage inclusively and that the history of the medical mistreatment of the Black patients highly impacted their thinking and perceptions (8/20 participants). Some examples are provided below (see Appendix Table S4):

“I liked learning about the topics when we broke out into groups and delved into the history…. it is unbelievable how Black individuals were treated, used for experiments and dehumanized. It is really going to make me change my thoughts and the way I approach individuals in my practice and in my everyday life!” (Participant 21)

“Having the opportunity to discuss race with a small group in a safe space was very helpful for me to gain new perspectives and I feel generally more comfortable with that type of discussion.” (Participant 12)

Participants also indicated that the training would lead them to take new actions in the future (see Appendix Table S6). Specifically, participants mentioned they would engage in “future conversations with either family, friends, or coworkers” (22/25 participants), “apply this experience to engage the broader community in conversations on race” (20/25 participants), “seek further outlets to learn more about how to build better race relations” (23/25 participants), and “integrate this [training] into treatment practices with patients” (22/25 participants). Only a few participants said that it was “too soon to apply this [training] experience in a practical way” (4/25 participants).

### Participant perception assessments

Table 1 presents results from questions about physician agreement with questions regarding patient relationships, comfort in talking about race, lived experiences of their Black patients, and racial disparities in OUD treatment. For each domain, the table shows (1) the proportion of participants who agreed with each item in the pre-training survey and the follow-up survey and (2) the percentage-point change in agreement from the pre-training survey to the follow-up survey.

**Table 1.** Proportion of participants in agreement with questions related to patient relationships, comfort in talking about race, patient lived experience, racial prejudice, and racial differences in treatment experiences in the pre-training and 2-month follow-up surveys.

Question description	Agree		Percentage-point change in agreement, pre to follow-up	*P*
	Pre	Follow-up		
Patient relationships				
I am able to connect on a personal level with Black/African American patients with opioid use disorder	40%	48%	0.08	.593
I am good at listening to Black/African American patients with opioid use disorder	92%	92%	0.00	1.000
I trust my Black/African American patients with opioid use disorder to follow through with their care plan	44%	52%	0.08	.564
My Black/African American patients with opioid use disorder trust me with their care	80%	72%	−0.08	.527
I never worry that I may be acting in a subtly prejudiced way toward Black/African American patients	28%	24%	−0.04	.739
Even though I know it's not appropriate, I sometimes feel that I hold unconscious negative attitudes toward Black/African American patients with opioid use disorder	8%	12%	0.04	.564
I worry that I have unconscious biases toward Black patients with opioid use disorder	20%	28%	0.08	.527
When treating Black/African American patients with opioid use disorder, I sometimes worry that I am unintentionally being prejudiced	24%	24%	0.00	1.000
I am completely comfortable talking about race…				
During training programs	52%	76%	0.24	.083
With family/friends	80%	88%	0.08	.480
With my patients	52%	52%	0.00	1.000
Other providers in my hospital	64%	72%	0.08	.564
Lived experience				
The lived experience of Black/African American patients with opioid use disorder is the same as patients of other races	4%	8%	0.04	.564
Have lived experiences similar to lived experiences of patients of other races	8%	16%	0.08	.414
I am interested in learning more about the lived experiences of Black/African American patients with opioid use disorder	96%	92%	−0.04	.564
Racism is a problem for the Black/African American community in the United States	84%	92%	0.08	.414
In Michigan Black patients with opioid use disorder…				
Adhere to treatment less than patients of other races	12%	12%	0.00	1.000
Are harder to treat than patients of other races	4%	4%	0.00	1.000
Are less likely to accept treatment than patients of other races	0%	4%	0.04	.317
Are more likely to discontinue treatment	16%	12%	−0.04	.705
Are more likely to overdose	8%	12%	0.04	.655
Have opioid use disorder at a similar level of severity compared to the severity of opioid use disorder among patients of other races	24%	16%	−0.08	.480
Receive similar treatment intensity as patients of other races	24%	20%	−0.04	.739
Receive the same scope of opioid use disorder treatments (eg, medications for opioid use disorder, psychotherapy, community mental health services) as patients of other races	20%	12%	−0.08	.414

Source: Authors’ analysis of New Detroit Just Care training evaluation data. Results report the proportion of agreement with each item asked in both the pre-survey and the 60-day follow-up survey. *P*-values compare differences in agreement over time for each instrument and were obtained using McNemar's test for paired nominal data.

### Patient relationships and implicit bias

In the baseline assessment survey, participants almost unanimously agreed that they were good at listening to their Black patients with OUD (92%) and that their Black patients trusted them (80%). Conversely, fewer than half of the participants agreed that they could connect personally with their Black patients with OUD (40%) and trusted that their Black patients would follow through with their care plans (44%). In the 2-month follow-up survey, 48% of participants said they were able to connect with their Black patients with OUD (48%; *P* = .593), while 52% of participants felt that Black patients would follow through with care (52%; *P* = .564). Meanwhile, the proportion of participants who felt that their Black patients trusted them with their care decreased to 72% (72%; *P* = .527).

In the baseline assessment survey, about one-quarter of participants felt concerned that they had an unconscious bias toward Black people with OUD (20%) and unintentionally treated patients with OUD prejudicially (24%), while slightly more than one-quarter never worried that they might be acting in a subtly prejudice manner towards their Black patients (28%). Further, less than 10% of participants experienced feeling negative attitudes toward Black patients with OUD (8%). In the 2-month follow-up survey, the proportion of participants who believed they never felt worried about being prejudiced toward Black patients decreased to 24% (24%; *P* = .739). The proportion of participants who felt concerned that they were unintentionally prejudiced (28%; *P* = .527) or held negative attitudes toward Black patients (12%; *P* = .564) also decreased.

Several participants also commented on increased awareness of their biases with their patients in the post-training survey:

“[The training] showed me that I likely have biases that I wasn't really aware of. While I may not be perfect overcoming them, I am going to take a step back with patients with OUD of color, and ask if I am treating them the appropriately.” (Participant 19)

In the 2-month follow-up survey, participants commented that the training helped them empathize and develop stronger relationships with patients:

“I allow more time to discuss and consider barriers to interface with the medical system and try to empathize with patients, specifically around factors influencing their OUD.” (Participant 11)

I have been trying to spend a few extra minutes developing a relationship letting them know they are being heard and trying to manage expectations from the beginning. (Participant 19)

### Comfort in discussing race

Provider comfort in discussing racial bias issues varied by whom they would consult with. In the baseline assessment survey, participants reported feeling more comfortable talking about racial bias in medicine with their family and friends (80%) and with other providers in their hospital (64%), while less comfortable talking about racial bias during training programs (52%) and with their patients (52%). However, in the 2-month follow-up survey, there was a 24-percentage-point increase in the proportion of participants who felt comfortable talking about racial bias during training programs (76%; *P* = .083) and 8-percentage-point increases in the proportion of participants who felt comfortable talking about racial bias with family/friends (88%; *P* = .480) and with other providers in their hospital (72%; *P* = .564).

### Lived experience of Black patients with OUD and unconscious bias awareness

Few participants agreed that the lived experiences of Black patients were the same as patients of other races in the baseline assessment survey (4%). This increased slightly to 8% in the follow-up (8%; *P* = .564). Almost all participants agreed that they were interested in learning more about the lived experiences of their Black patients with OUD (96%). However, there was a slight decrease in interest in the 2-month follow-up survey (92%; *P* = .564). More than 80% described racism as a problem for Black communities (84%) in the baseline assessment survey, which increased to 92% in the 2-month follow-up survey (92%; *P* = .414).

In post-training survey questions, participants indicated that the training was instrumental in understanding racial disparities in patients’ lived experiences (see Appendix Table S4). One participant commented:

“I think there is a constant struggle to understand the context of the disparities in health outcomes we see in the ED every day. [The training] raised my awareness and empathy towards the lived experiences of my patients from disadvantaged populations.” (Participant 8)

### Changes in treatment disparities perceptions

In the baseline assessment survey, only a few participants agreed that Black patients with OUD may be less likely to adhere to treatment than patients of other races (12%), were more challenging to treat than patients of other races (4%), and were more likely to overdose (8%) or discontinue treatment (16%). No participants agreed that Black patients were less likely to accept treatment (0%). About one-quarter of participants agreed that, as compared with patients of other races, OUD severity (24%), treatment intensity (24%), and scope of treatment (20%) for Black patients were similar. In the baseline assessment survey open-response questions, several participants said they had not witnessed Black patients receiving less care, while others provided direct examples of discriminatory behavior (see Appendix Table S3).

For instance, 1 provider commented:

“I see colleagues and staff react differently toward non-White patients with opioid use disorder. Even those who are trying to overcome their addiction with methadone or suboxone.” (Participant 2)

While another said:

“I treat every patient who comes in with OUD the same. I have had patients of all races be kind and willing to go to treatment and I have had others who will scream, demand pain medication, and be rude to staff. I don't see race play into it.” (Participant 9)

In the 2-month follow-up survey, there were 4-percentage-point increases in the proportion of participants who believed Black patients were less likely to accept treatment (4%; *P* = .317) and more likely to overdose (12%; *P* = .655), 4-percentage-point decreases in the proportion of participants who believed that Black patients were more likely to discontinue treatment (12%; *P* = .705) or receive similar treatment intensity (20%; *P* = .739), and 8-percentage-point decreases in the proportion of participants who perceived similarity in patient OUD severity (16%; *P* = .480) and scope of treatment (12%; *P* = .414).

### Differences in perceptions of Black and White patients with OUD in the ED

Table 2 presents results on the perceptions of participants towards Black and White patients with OUD whom they treated. In the post-training survey, differences in the perceptions of Black vs White patients by participants were most prominent for drug-seeking behaviors (12% Black and 24% White), frustration in taking care of (16% Black and 28% White), satisfaction to take care of (32% Black and 24% White), failure to comply with medical advice (28% Black and 24% White), made participants glad they went into medicine (32% Black and 24% White), manipulates providers (16% Black and 24% White), and over-exaggerated pain (8% Black and 20% White).

**Table 2.** Implicit bias towards patients with OUD in emergency departments—proportion of participants agreeing that more than half their patients fall into a specific category.

	>50%						Percentage-point change in Black–White differences post to follow-up	*P*
	Post			Follow-up				
	Black	White	Dif	Black	White	Dif		
What proportion of patients with OUD…								
Are drug-seeking when they come to the hospital	12%	24%	−0.12	8%	20%	−0.12	0.00	.655
Are easy to empathize with	32%	32%	0	40%	32%	0.08	0.08	.180
Are frustrating to take care of	16%	28%	−0.12	12%	16%	−0.04	0.08	.102
Are satisfying to take care of	32%	24%	0.08	36%	36%	0	−0.08	.480
Are the kind of person I could see myself being friends with	12%	12%	0	8%	8%	0	0.00	.317
Fail to comply with medical advice	28%	24%	0.04	20%	20%	0	−0.04	.655
Makes me glad that I went into medicine	32%	24%	0.08	40%	32%	0.08	0.00	.414
Manipulate you or other providers	16%	24%	−0.08	12%	20%	−0.08	0.00	.257
Over-report -exaggerate- pain	8%	20%	−0.12	8%	12%	−0.04	0.08	.655

	Pre: agree			Follow-up: agree			Percentage-point change in Black–White differences pre to follow-up	
	Black	White	Dif	Black	White	Dif		
Patients with OUD often…								
Talking on the phone or watching TV, while complaining of severe pain	40%	40%	0	24%	40%	−0.16	−0.16	.157
Change their behavior—eg, appears in great distress—when a provider walks into the room	52%	44%	0.08	24%	40%	−0.16	−0.24	.206
Have a dispute with the staff	48%	44%	0.04	16%	24%	−0.08	−0.12	.257
Request a specific narcotic drug and dose	40%	56%	−0.16	36%	48%	−0.12	0.04	.705
Ring the bell for the nurse and ask for more pain medication before the next dose is due	48%	52%	−0.04	28%	36%	−0.08	−0.04	.655
Sign out against medical advice	36%	36%	0	24%	36%	−0.12	−0.12	.705
Tamper with an IV, PICC line, or tamper with patient-controlled analgesia devices	4%	8%	−0.04	8%	12%	−0.04	0.00	.317

Abbreviations: Dif, the percentage point difference in agreement between black and white respondents; IV, intravenous; OUD, opioid use disorder; PICC, peripherally inserted central catheter.

Source: Authors’ analysis of New Detroit Just Care training evaluation data. Results report the proportion of agreement with each item asked in both the post-training survey (ie, the survey delivered immediately after the training and the 60-day follow-up survey). *P*-values compare Black–White differences in agreement over time for each instrument and were obtained using McNemar's test for paired nominal data.

Differences between the post-training and 2-month follow-up survey differences increased for Black patients for easy to empathize with (8 percentage points; *P* = .180), frustration taking care of (8 percentage points; *P* = .102), and exaggerated pain (8 percentage points; *P* = .655). Further, differences decreased for Black patients in satisfied to take care of (8 percentage points; *P* = .480) and failing to comply with medical advice (4 percentage points; *P* = .655).

In the second domain, participants were asked about their agreement with certain drug-seeking behaviors that patients with OUD might engage in by race. In the post-survey, participants were more likely to agree that their Black patients would change their behavior when a provider walks in the room (52% Black and 44% White) or have a dispute with staff (48% Black and 44% White). Participants were less likely to agree that, relative to White patients, Black patients requested specific narcotics (40% Black and 56% White), requested more medications before the next dose was due (48% Black and 52% White), and tampered with patient-controlled analgesia devices (4% Black and 8% White). Differences between post-training and 2-month follow-up survey differences were almost always negative (ie, Black patients were viewed as less likely to engage in drug-seeking behaviors than White patients over time).

### Changes in knowledge of topics covered in the training

In Table 3, we report responses to questions that gauged provider knowledge of topics covered in the training. In the baseline assessment, about two-thirds of participants were familiar with all concepts, which included the 1980s war on drugs (68%), social determinants of health (60%), Drug Enforcement Administration classification of narcotics (68%), historical mistrust of the health care system by the Black community (64%), implicit bias (72%), and the Tuskegee experiment (56%). Familiarity with concepts increased in the post-training survey: 20 percentage points for the 1980 war on drugs (88%; *P* = .059), 28 percentage points for social determinants of health (88%; *P* = .035), 16 percentage points for the Drug Enforcement Administration classification of narcotics (84%; *P* = .206), 8 percentage points for historical mistrust of the health care system within the Black community (72%; *P* = .527), 20 percentage points for implicit bias (92%; *P* = .059), and 32 percentage points for the Tuskegee experiment (88%; *P* = .011).

**Table 3.** Proportion of provider familiarity with key concepts on racial discrimination in medicine covered in the New Detroit Just Care racial bias training.

Question description	Pre: familiar	Post: familiar	Percentage-point change pre to post	*P*
How familiar are you with…				
1980s War on Drugs	68%	88%	0.20	.059
Concept of social determinants of health	60%	88%	0.28	.035
DEA classification of narcotics	68%	84%	0.16	.206
Historical mistrust of the health care, social services, and the justice system in the Black/African American community	64%	72%	0.08	.527
Term implicit bias	72%	92%	0.20	.059
Tuskegee experiment	56%	88%	0.32	.011

Abbreviation: DEA, Drug Enforcement Administration.

Source: Authors’ analysis of New Detroit Just Care training evaluation data. Results report the proportion of agreement with each item asked in both the pre-training survey and post-training survey. *P*-values compare differences in participants responding “familiar” over time for each instrument and were obtained using McNemar's test for paired nominal data.

In the post-training survey, several participants emphasized that historical context helped them to understand the potential for Black patients to mistrust the medical community (see Appendix Table S4):

I gained a lot of knowledge about the history of Blacks/African Americans and… how they were mistreated…. It helped gain a new perspective on the medical mistrust in Black/African American communities which, in turn, will help me be a better provider. (Participant 12)

Table 4 summarizes provider responses to treatment disparities and outcomes-related questions about racial disparities in OUD and questions specific to the Michigan context in the baseline assessment and post-training survey. Most participants stated that they believed that White patients were more likely to receive medications for OUD (80%), that Black individuals with OUD had a higher overdose death rate than White individuals (72%), and that White pregnant patients with OUD were more likely to receive buprenorphine than Black pregnant patients (68%). Fewer participants responded that treatment retention for Black patients was higher than for White patients (4%) and that overdose deaths were increasing faster for White people who use drugs than for Black people (36%).

**Table 4.** Treatment and health outcomes disparities knowledge assessment: proportion of participants who responded “higher” to each question.

Question description	Pre: higher	Post: higher	Percentage-point change pre to post	*P*
Generally, White patients are at a ____ likelihood to receive medications for OUD—eg, buprenorphine—than/as Black patients	80%	96%	0.16	.102
In Michigan, the opioid overdose death rate among the Black population—ie, per 100 000 residents—is ______ than the opioid overdose death rate in the White population	72%	84%	0.12	.317
Non-White patients are at a ______ likelihood to be retained beyond 6 months in buprenorphine treatment compared with White patients	4%	12%	0.08	.157
The rate of synthetic opioid overdose deaths is increasing at a ______ rate for White people who use drugs than Black people who use drugs	36%	20%	−0.16	.206
White pregnant patients with OUD are at a ______ likelihood to receive buprenorphine treatment than Black pregnant patients with OUD	68%	88%	0.20	.132

Abbreviation: OUD, opioid use disorder.

Source: Authors’ analysis of New Detroit Just Care training evaluation data. Results report the proportion of agreement with each item asked in both the pre-training survey and post-training survey. *P*-values compare differences in those that responded “higher” over time for each instrument and were obtained using McNemar's test for paired nominal data.

Provider acknowledgment of disparities in treatment and outcomes increased in the post-training survey. Specifically, the proportion of participants who said that White patients were more likely to get medications for OUD increased by 16 percentage points (96%; *P* = .102), by 12 percentage points for the proportion who said the overdose death rates of Black Americans were higher than those of White Americans (84%; *P* = .317), and by 20 percentage points for the proportion who said that White pregnant patients with OUD were more likely to receive buprenorphine (88%; *P* = .132). Further, there was a 16-percentage-point decrease in the proportion of participants who believed that the synthetic opioid overdose death rate was increasing faster for White people who use drugs (20%; *P* = .206).

### Changes in treatment practices

Table 5 presents responses to questions about provider treatment practices in the baseline assessment survey and 2-month follow-up survey. Nearly three-quarters (72%) of participants stated that Black patients represented greater than half of the patients they encountered with OUD in a typical month, 32% said that Black patients were more than half of the patients they followed up with after discharge, 64% said more than half of the patients that they referred to follow-up outpatient care were Black, and 56% said that more than half of the patients to whom they prescribed buprenorphine were Black.

**Table 5.** The proportion of participants who responded that more than half their Black patients receive a specific type of treatment.

Question description	>50%		Percentage-point change pre to post	*P*
	Pre	Follow-up		
What proportion of OUD patients that you encounter in a typical month are Black/African American	72%	60%	−0.12	.317
What proportion of patients that you or your team follow-up with post-discharge in a typical month are Black/African American	32%	44%	0.12	.317
What proportion of patients that you refer to follow-up care—warm hand-offs—in a typical month are Black/African American	64%	64%	0.00	1.000
What proportion of patients whom you treat with buprenorphine in a typical month are Black/African American	56%	48%	−0.08	.564

Abbreviation: OUD, opioid use disorder.

Source: Authors’ analysis of New Detroit Just Care training evaluation data. Results report the proportion of agreement with each item asked in both the pre-training survey and the 60-day follow-up survey. *P*-values compare differences in those that responded “>50%” over time for each instrument and were obtained using McNemar's test for paired nominal data.

In the post-training survey, 60% and 8% of participants responded that more than half of their encounters and buprenorphine prescriptions were for Black patients, respectively (60% and 8%; *P* = .317 and *P* = .564). However, there was a 12-percentage-point increase in the proportion of participants who stated that more than half of the patients they followed up with were Black (44%; *P* = .317).

## Discussion

### Summary of findings

Overall, participants reported strong satisfaction with the training. All participants responded to all 3 of the surveys with high fidelity and participated throughout the training. Participants found discussions of historical inequities in the delivery of medical services to Black patients highly informative and the small-group discussions especially meaningful. Participants were also notably impacted by the discussions about the lived experiences of Black patients with OUD and the motivational interviewing component, as evidenced by qualitative responses in the post-training and 2-month follow-up surveys. Participants further described an appreciation for having a safe environment to discuss racial disparities in OUD treatment in their EDs and found that the training promoted racial healing. Findings indicate the feasibility and acceptability of the pilot training and suggest the potential for scaling the training to larger groups and more health care organizations in future evaluations of training effectiveness.

Although this study is unable to draw generalizable conclusions or causal inferences regarding the effectiveness of the training due to the small sample size, the few hospitals from which participants were drawn, and the lack of randomization, there were some preliminary results worth mentioning. First, at baseline, some participants acknowledged disparities in treatment and outcomes for their patients with OUD and exhibited some familiarity with concepts covered in the training, like implicit bias. However, after the training, participants indicated that they were more likely to recognize disparities in OUD care delivery and outcomes. There were also some indications that participants gained new knowledge of key concepts and information covered during the training, given 2 results having a *P*-value of less than .05. However, using Bonferroni correction, only items with a *P*-value below .001 should be considered statistically significant (ie, .05 alpha/53 items), and both *P*-values for these items were larger than that threshold. Moreover, although no significant changes in provider practice were observed by the time of the 2-month follow-up survey, providers indicated they intended to integrate knowledge obtained from the training into practice.

### Implications for racial bias in OUD training program design

Given the satisfaction of participants and responses indicating its usefulness, our results suggest that New Detroit's Just Care training should be considered for expansion and additional evaluation. Although larger effectiveness studies are needed to confirm the effect of the training on perceptions and practice, we found that the New Detroit training anecdotally improved providers’ awareness of issues surrounding treatment and outcome inequalities inherent in the US opioid epidemic and the field of medicine. Michigan's requirement of racial bias training as a condition of medical licensure will expand the number of providers receiving racial bias training, and this pilot evaluation offers some guidance on how to design training to ensure participant acceptability.

Direct engagement between participants and facilitators was cited by nearly all participants as being the most impactful on their perceptions and understanding of inequity in OUD service delivery. Ensuring that trainings have sufficient small-group breakouts, motivational interviewing components, and staff–participant engagement may be important to maximizing benefits in subsequent trainings. Moreover, providing detailed historical contexts of the mistreatment of Black patients was associated with an increased understanding of the potential hesitance toward health care systems among their patients. Including historical key context in future and related trainings may help providers engage more effectively with their patients. Although participants were generally familiar with the concept of implicit bias at baseline, many had not recognized their own implicit biases. Self-reflection exercises were anecdotally effective in driving greater self-awareness in participants. Motivational interviewing was also cited as one of the more impactful training components. To measure training component effectiveness, larger scale implementation with randomization will be needed. Results concerning changes (or the lack thereof) in equitable care delivery could then be linked to patient health (eg, opioid overdose rates among patients treated by participants) and medical spending outcomes (eg, savings obtained by more equitable care delivery) to measure the value of the New Detroit training program.

### Limitations

There are several study limitations. First, all data were self-reported survey responses and may include desirability bias given the nature of the material. Second, only 25 providers participated in the training across 2 hospitals, limiting the generalizability of our findings outside of these hospitals and participants. Third, providers volunteered to participate in the training, so that selection may bias results; participants may differ in their perceptions from nonparticipants. Finally, the small number of participants in the survey likely prevented us from detecting statistical significance in changes in practice and perceptions over time; a sample of 25 is close to the minimum needed to detect statistical significance with McNemar's test. More participants will be needed to confirm the strength and significance of the associations described above, and a broader set of hospitals across geographic regions will be required to ensure sufficient generalizability.

## Conclusion

The New Detroit Just Care racial equity training delivered to emergency medicine providers in southeast Michigan hospitals focused on educating providers on implicit bias in medicine and OUD treatment, increasing knowledge of racial disparities in OUD treatment and outcomes, and increasing comfort in discussing race with others. The training participants indicated satisfaction with the training, were engaged in the material during the training, and responded to all survey questions and components. Together, this implies the feasibility and acceptability of scaling the pilot training to more OUD treatment providers in Michigan. In addition, our study found historical information on racial inequity in medical care, motivational interviewing, and small-group discussion sections to be the most meaningful training components for participants. These training components should likely be emphasized as the New Detroit training and others like it begin to scale in response to Michigan legislation requiring implicit bias training among health care providers.

## Supplementary Material

qxae049_Supplementary_Data

## References

[1] Gopal DP , Chetty U, O’Donnell P, Gajria C, Blackadder-Weinstein J. Implicit bias in healthcare: clinical practice, research and decision making. Future Healthc J. 2021;8(1):40–48. 10.7861/fhj.2020-0233 33791459 PMC8004354

[2] Marcelin JR , Siraj DS, Victor R, Kotadia S, Maldonado YA. The impact of unconscious bias in healthcare: how to recognize and mitigate it. J Infect Dis. 2019;220(Suppl 2):S62–S73. 10.1093/infdis/jiz214 31430386

[3] Sabin JA . Tackling implicit bias in health care. N Engl J Med. 2022;387(2):105–107. 10.1056/NEJMp2201180 35801989 PMC10332478

[4] Sabin JA , Greenwald AG. The influence of implicit bias on treatment recommendations for 4 common pediatric conditions: pain, urinary tract infection, attention deficit hyperactivity disorder, and asthma. Am J Public Health. 2012;102(5):988–995. 10.2105/AJPH.2011.300621 22420817 PMC3483921

[5] Smedley B , Stith A, Nelson A. Unequal Treatment: Confronting Racial and Ethnic Disparities in Health Care. National Academies Press; 2003:12875. 10.17226/12875 25032386

[6] Sun M , Oliwa T, Peek ME, Tung EL. Negative patient descriptors: documenting racial bias in the electronic health record: study examines racial bias in the patient descriptors used in the electronic health record. Health Aff (Millwood). 2022;41(2):203–211. 10.1377/hlthaff.2021.01423 35044842 PMC8973827

[7] Williams DR , Mohammed SA. Discrimination and racial disparities in health: evidence and needed research. J Behav Med. 2009;32(1):20–47. 10.1007/s10865-008-9185-0 19030981 PMC2821669

[8] Zeidan AJ , Khatri UG, Aysola J, et al Implicit bias education and emergency medicine training: step one? Awareness. AEM Educat Train. 2019;3(1):81–85. 10.1002/aet2.10124 PMC6339553 30680351

[9] Zestcott CA , Blair IV, Stone J. Examining the presence, consequences, and reduction of implicit bias in health care: a narrative review. Group Processes Intergroup Relat. 2016;19(4):528–542. 10.1177/1368430216642029 PMC4990077 27547105

[10] Parlier-Ahmad AB , Pugh M, Martin CE. Treatment outcomes among Black adults receiving medication for opioid use disorder. J Racial Ethn Health Disparities. 2022:9(4):1557–1567. 10.1007/s40615-021-01095-4 34254271 PMC8274965

[11] Michigan Department of Health and Human Services . Age-adjusted opioid drug overdoses mortality rates by sex, Black Michigan residents 2000–2022. 2022. Accessed February 7, 2024. https://www.mdch.state.mi.us/osr/fatal/BH1fBlackRates.asp

[12] Gibbons JB , McCullough JS, Brown ZY, Zivin K, Norton EC. Racial and ethnic disparities in medication for opioid use disorder access, use, and treatment outcomes in Medicare. J Subst Use Addic Treat. 2024;157:209271. 10.1016/j.josat.2023.209271 38135120

[13] Gibbons JB , Harris SJ, Soloman K, Sugarman OK, Hardy C, Saloner B. The rising tide of overdose deaths among Black Americans: a review of the literature. Lancet Psychiatry. 2022;10(9):719–726. 10.1016/S2215-0366(23)00119-0 37236218

[14] Hagiwara N , Kron FW, Scerbo MW, Watson GS. A call for grounding implicit bias training in clinical and translational frameworks. Lancet. 2020;395(10234):1457–1460. 10.1016/S0140-6736(20)30846-1 32359460 PMC7265967

[15] Cooper LA , Saha S, Van Ryn M. Mandated implicit bias training for health professionals—a step toward equity in health care. JAMA Health Forum. 2022;3(8):e223250. 10.1001/jamahealthforum.2022.3250 36218984

[16] Maina IW , Belton TD, Ginzberg S, Singh A, Johnson TJ. A decade of studying implicit racial/ethnic bias in healthcare providers using the implicit association test. Soc Sci Med. 2018;199:219–229. 10.1016/j.socscimed.2017.05.009 28532892

[17] Nelson A . Unequal treatment: confronting racial and ethnic disparities in health care. J Natl Med Assoc. 2022;94(8):666–668. PMC2594273 12152921

[18] Pennington CR , Bliss E, Airey A, Bancroft M, Pryce-Miller M. A mixed-methods evaluation of unconscious racial bias training for NHS senior practitioners to improve the experiences of racially minoritised students. BMJ Open. 2023;13(1):e068819. 10.1136/bmjopen-2022-068819 PMC9872466 36669838

[19] Ruben M , Saks NS. Addressing implicit bias in first-year medical students: a longitudinal, multidisciplinary training program. Med Sci Educ. 2020;30(4):1419–1426. 10.1007/s40670-020-01047-3 34457809 PMC8368581

[20] Castillo LG , Brossart DF, Reyes CJ, Conoley CW, Phoummarath MJ. The influence of multicultural training on perceived multicultural counseling competencies and implicit racial prejudice. J Multicult Couns Devel. 2007;35(4):243–255. 10.1002/j.2161-1912.2007.tb00064.x

[21] Sherman MD , Ricco J, Nelson SC, Nezhad SJ, Prasad S. Implicit bias training in residency program: aiming for enduring effects. Fam Med. 2019;51(8):677–681. 10.22454/FamMed.2019.947255 31509218

[22] Steed R . Cultural competency instruction in a 3D virtual world (no. 305150297). Accessed February 7, 2024. https://www.proquest.com/dissertations-theses/cultural-competency-instruction-3d-virtual-world/docview/305150297/se-2

[23] Community Foundation for Southeast Michigan. Equitable Care That Meets People Where They Are in Their Journey. Accessed May 3, 2024. https://cfsem.org/initiative/opioid/our-work/

[24] Michigan Department of Licensing and Regulatory Affairs . Proposed rules. 2020. Accessed February 7, 2024. https://www.michigan.gov/documents/lara/LARA_PHC_Rules_Text_704173_7.pdf

[25] Haywood C , Lanzkron S, Hughes MT, et al A video-intervention to improve clinician attitudes toward patients with sickle cell disease: the results of a randomized experiment. J Gen Intern Med. 2011;26(5):518–523. 10.1007/s11606-010-1605-5 21181560 PMC3077483

[26] Katz AD , Hoyt WT. The influence of multicultural counseling competence and anti-Black prejudice on therapists’ outcome expectancies. J Couns Psychol. 2014;61(2):299–305. 10.1037/a0036134 24635592

[27] Bauer S & Bell S. Detroit overdose surveillance, 2012–2020. 2021. Accessed February 7, 2024. https://detroitmi.gov/sites/detroitmi.localhost/files/2023-04/UPDATED%202021%20Report%20%28Detroit%20Overdose%20Surveillance%202012-2020%29.pdf

[28] US Census Bureau . Quick facts—Detroit city, Michigan; United States. 2022. Accessed February 7, 2024. https://www.census.gov/quickfacts/fact/table/detroitcitymichigan,US/PST045222

