# Supplementary material for: Piloting racial bias training for hospital emergency department providers treating patients with opioid use disorder
Source: Health Aff Sch. 2024 Apr 24;2(5):qxae049. doi: 10.1093/haschl/qxae049 (PMC11095526; doi:10.1093/haschl/qxae049)

**Supplementary Appendix**

**Appendix Table 1. Questions from Just Care Evaluation Baseline, Immediate Post, and Follow-up Training Surveys**

| Question | Baseline | Immediate Post-Training | 2-Month Post Assessment |
| --- | --- | --- | --- |
| Provider Demographics | | | |
| 1. *Please select the year you were born from the dropdown menu.* | X |  |  |
| 1. *What gender do you most identify with?* | X |  |  |
| 1. *Please indicate your race.* | X |  |  |
| 1. *Are you of Hispanic or Latino origin?* | | | |
| 1. *Provider Practice Characteristics* | | | |
| 1. *Please select your role.* | X |  |  |
| 1. *Please select your primary practice setting.* | X |  |  |
| 1. *Please note your secondary practice setting, if applicable.* | X |  |  |
| 1. *How many years have you been practicing in the Emergency Department?* | X |  |  |
| 1. *Do you have a buprenorphine waiver?* | X |  |  |
| 1. *If yes, what year did you obtain a buprenorphine waiver?* | X |  |  |
| 1. *Have you ever/in the past 2 months have you treated a patient overdosing on opioids?* | X |  | X |
| 1. *Have you ever/in the past 2 months have you prescribed buprenorphine to an ED patient with OUD?* | X |  | X |
| 1. *Have you ever/in the past 2 months have you inducted a patient on buprenorphine in the ED?* | X |  | X |
| 1. *Have you ever previously completed racial bias training?* | X |  |  |
| 1. *If yes, what year did you complete the racial bias training?* | X |  |  |
| 1. *Have you ever previously completed addiction stigma training?* | X |  |  |
| 1. *If yes, what year did you complete the addiction stigma training?* | X |  |  |
| Racial Bias Awareness and Perceptions | | | |
| 1. Please rank your level of agreement with the following statements… | | | |
| *Even though I know it’s not appropriate, I sometimes feel that I hold unconscious negative attitudes toward Black/African American patients with opioid use disorder.* | X |  | X |
| *When treating Black/African American patients with opioid use disorder, I sometimes worry that I am unintentionally being prejudiced.* | X |  | X |
| *I worry that I have unconscious biases toward Black/African American patients with opioid use disorder.* | X |  | X |
| *I never worry that I may be acting in a subtly prejudiced way toward Black/African American patients.* | X |  | X |
| *My Black/African American patients with opioid use disorder trust me with their care.* | X |  | X |
| *I trust my Black/African American patients with opioid use disorder to follow through with their care plan.* | X |  | X |
| *I am good at listening to Black/African American patients with opioid use disorder.* | X |  | X |
| *I am able to connect on a personal level with Black/African American patients with opioid use disorder.* | X |  | X |
| *I am interested in learning more about the lived experiences of Black/African American patients with opioid use disorder.* | X |  | X |
| 1. What proportion of [race] patients with opioid use disorder:   [Questions asked for Black/African American and White patients separately] | | | |
| *Over-report (exaggerate) pain?* | X |  | X |
| *Fail to comply with medical advice?* | X |  | X |
| *Manipulate you or other providers?* | X |  | X |
| *Are drug-seeking when they come to the hospital?* | X |  | X |
| *Are frustrating to take care of?* | X |  | X |
| *Makes me glad that I went into medicine?* | X |  | X |
| *Are the kind of person I could see myself being friends with?* | X |  | X |
| *Are satisfying to take care of?* | X |  | X |
| *Are easy to empathize with?* | X |  | X |
| 1. [Race] patients with opioid use disorder often:   [Questions asked for Black/African American and White patients separately] | | | |
| *Request a specific narcotic drug and dose* | X |  | X |
| *Appear comfortable (e.g., talking on the phone or watching TV) while complaining of severe pain* | X |  | X |
| *Have a dispute with staff* | X |  | X |
| *Ring the bell for nurse and ask for more pain medication before next dose is due* | X |  | X |
| *Change their behavior (e.g., appears in great distress) when a provider walks in the room* | X |  | X |
| *Sign out against medical advice* | X |  | X |
| *Tamper with an IV, PICC line, or tamper with patient-controlled analgesia devices* | X |  | X |
| Comfort in talking about issues related to implicit bias, race, disparities | | | |
| 1. Please rank your level of agreement with the following statements… | | | |
| *I am completely comfortable talking about race with family/friends.* | X | X | X |
| *I am completely comfortable talking about race with other providers in my hospital.* | X | X | X |
| *I am completely comfortable talking about race with my patients.* | X | X | X |
| *I am completely comfortable talking about race during training programs.* | X | X | X |
| *Racism is a problem for the Black/African American community in the US.* | X | X | X |
| *The lived experience of Black/African American patients with opioid use disorder is the same as patients of other races.* | X | X | X |
| Knowledge of history of racism in medicine and history of opioid epidemic | | | |
| 1. How familiar are you with the… | | | |
| *Historical mistrust of the health care, social services, and the justice system in the Black/African American community?* | X | X |  |
| *Tuskegee Experiment?* | X | X |  |
| *Term “Implicit Bias”* | X | X |  |
| *1980’s War on Drugs?* | X | X |  |
| *Concept “social determinants of health”* | X | X |  |
| *DEA classification of narcotics?* | X | X |  |
| Disparities in opioid use disorder treatment and outcomes | | | |
| 1. In Michigan, Black/African American patients with opioid use disorder… | | | |
| *Receive similar treatment intensity as patients of other races* | X | X | X |
| *Receive the same scope of opioid use disorder treatments (i.e., medications for opioid use disorder, psychotherapy, community mental health services) as patients of other races* | X | X | X |
| *Have lived experiences similar to lived experiences of patients of other races* | X | X | X |
| *Have opioid use disorder at a similar level of severity compared to the severity of opioid use disorder among patients of other races* | X | X | X |
| *Are harder to treat than patients of other races* | X | X | X |
| *Are less likely to accept treatment than patients of other races* | X | X | X |
| *Adhere to treatment less than patients of other races* | X | X | X |
| *Are more likely to overdose than patients of other races* | X | X | X |
| *Are more likely to discontinue treatment than patients of other races* | X | X | X |
| Fill in the blanks in the following statements: | | | |
| 1. *In Michigan, the opioid overdose death rate among the Black population (i.e., per 100,000 residents) is lower than/equal to/higher than the opioid overdose death rate in the White population.* | X | X |  |
| 1. *Generally, White patients are less likely/equally likely/more likely to receive medications for opioid use disorder (e.g., buprenorphine) than/as Black patients.* | X | X |  |
| 1. *The rate of synthetic opioid overdose deaths is increasing at a slower rate/equal rate/higher rate for White people who use drugs than Black people who use drugs.* | X | X |  |
| 1. *White pregnant patients with opioid use disorder are less likely/equally likely/more likely to receive buprenorphine treatment than Black pregnant patients with opioid use disorder.* | X | X |  |
| 1. *Non-White patients are ______ to be retained beyond six months in buprenorphine treatment compared with White patients.* | X | X |  |
| True/False: |  |  |  |
| 1. *Generally, US counties with large Black and Hispanic/Latino communities have more facilities to provide buprenorphine per capita, while counties with large White communities have more facilities to provide methadone per capita.* | X | X |  |
| Personal Experiences | | | |
| 1. *Have you ever witnessed treatment of Black/African American patients getting opioid use disorder treatment/treatment not in line with best practices? Please explain your choice in the space provided below.* | X |  |  |
| 1. *Have you ever witnessed racial prejudice in job promotions/positions at your facility? Please explain your choice in the space provided below.* | X |  |  |
| 1. *Have you ever witnessed Black/African American opioid use disorder patients being talked to in a disrespectful way? Please explain your choice in the space provided below.* | X |  |  |
| 1. *Have you ever witnessed Black/African American patients having opioid use disorder symptoms ignored by other doctors (i.e., pain, withdrawal, etc.)? Please explain your choice in the space provided below.* | X |  |  |
| Provider Practice Behaviors | | | |
| 1. *How many patients with opioid use disorder do you encounter in a typical month?* | X |  | X |
| 1. *What proportion of opioid use disorder patients that you encounter in a typical month are Black/African American?* | X |  | X |
| 1. *How many patients with opioid use disorder do you treat with buprenorphine in a typical month* | X |  | X |
| 1. *What proportion of patients that you treat with buprenorphine in a typical month are Black/African American?* | X |  | X |
| 1. *How many patients with opioid use disorder do you refer to follow-up care ("warm hand-offs") in the community in a typical month?* | X |  | X |
| 1. *What proportion of patients that you refer to follow-up care ("warm hand-offs") in a typical month are Black/African American?* | X |  | X |
| 1. *How many patients with opioid use disorder do you and your team follow-up with post discharge in a typical month?* | X |  | X |
| 1. *What proportion of patients that you and your team follow-up with post discharge in a typical month are Black/African American?* | X |  | X |
| Training Reflections | | | |
| 1. Based on my experience with this conversation on race, I am considering the following actions (Check all that apply) | | | |
| *Engage in future conversations with either family, friends, or co-workers* |  | X |  |
| *Apply this experience to engage the broader community in conversation on* |  | X |  |
| *Seek further outlets to learn more about how to build better race relations* |  | X |  |
| *Integrate this into my treatment practices with patients* |  | X |  |
| *Too soon to apply this experience in a practical way* |  | X |  |
| *Was this session useful to help you to communicate your own perceptions about race?* |  | X |  |
| *Was this session useful to help you to understand someone else’s perceptions about race? Please explain your choice in the space provided below.* |  | X |  |
| *What new concepts and knowledge did you obtain from this session?* |  | X |  |
| *Did the training provide timely information or new insights relevant to your work? Why or why not?* |  | X |  |
| *Did the training provide timely information or new insights relevant to your personal life? Why or why not?* |  | X |  |
| 1. *In the past 2 months, have you witnessed racial prejudice in job promotions/positions at your facility? Please explain your choice in the space provided below.* |  |  | X |
| 1. *In the past 2 months, have you witnessed treatment of Black/African American patients getting opioid use disorder treatment/treatment not in line with best practices? Please explain your choice in the space provided below.* |  |  | X |
| 1. *In the past 2 months, have you witnessed Black/African American opioid use disorder patients being talked to in a disrespectful way? Please explain your choice in the space provided below.* |  |  | X |
| 1. *In the past 2 months, have you witnessed Black/African American patients having opioid use disorder symptoms ignored by other doctors (i.e., pain, withdrawal, etc.)? Please explain your choice in the space provided below.* |  |  | X |
| 1. *Did attending the training impact your awareness of equitable treatment of Black/African American patients with opioid use disorder in your workplace? Please explain your choice in the space provided below.* |  |  | X |
| 1. *Did attending the training impact how you interacted with Black patients with opioid use disorder in the past 2 months? Please explain your choice in the space provided below.* |  |  | X |
| 1. *What specific needs are there to better serve Black patients with opioid use disorder in your ED?* |  |  | X |
| 1. *What specific needs are there to improve racial equity in health services in your community?* |  |  | X |
| 1. *What stuck with you the most from the training?* |  |  | X |
| 1. *Michigan is now requiring racial equity training for emergency department practitioners. Do you think other states should require this training for their emergency department practitioners? Please explain your choice in the space provided below.* |  |  | X |

**Appendix Table 2. Characteristics of Providers Participating in the New Detroit Just Care Racial Equity Training Program**

| **Variable** | **Value** |
| --- | --- |
| **Total Participants** | 25 |
| **Average Age** | 39.6 |
| **Race** |  |
| White | 22 (88%) |
| Black | 1 (4%) |
| Multiracial | 1 (4%) |
| Other/Unknown | 1 (4%) |
| **Ethnicity** |  |
| Hispanic | 1 (4%) |
| Non-hispanic | 24 (96%) |
| **Gender** |  |
| Female | 11 (44%) |
| Male | 14 (56%) |
| **Years Working in ED (Mean, STD)** | 12.4 (8.7) |
| **Provider type (N, %)** |  |
| Emergency Medicine Physician | 16 (64%) |
| Physician Assistant | 9 (36%) |
| **Buprenorphine Characteristics (N, %)** |  |
| Buprenorphine Waivered | 22 (88%) |
| Prescribed Buprenorphine in the ED | 18 (72%) |
| Inducted a Patient on Buprenorphine in the ED | 17 (68%) |
| **Treated an Overdose (N, %)** | 23 (92%) |
| **Racial Bias Training (N, %)** |  |
| Received Racial Bias Training | 15 (60%) |
| Received Addiction Stigma Training | 7 (28%) |

Notes: Authors’ analysis of New Detroit Just Care training evaluation data.

**Appendix Table 3. Summary of major themes and subthemes from the baseline assessment survey, New Detroit Just Care Training participants (n=25), 2022**

| **Theme** | **Illustrative quote** |
| --- | --- |
| **Have you ever witnessed treatment of Black/African American patients getting opioid use disorder treatment/treatment not in line with best practices? (n= 7)** | |
| Yes, patients are treated differently because they are Black or because they have OUD | *I see colleagues and staff react differently toward non-white patients with opioid use disorder. Even those who are trying to overcome their addiction with methadone or suboxone.* -Participant 2  *I have seen providers not comfortable starting medications for OUD for various reasons (COWS score not high enough, not trained, do not feel that they will be compliant). As I practice in a predominately Black/AA community, I can assume some of these patients statistically were black/aa however if they are not my patient, I am not certain what their race is.* -Participant 23 |
| No, patients receive equal treatment regardless of race | *I treat every patient who comes in with OUD the same. I have had patients of all races be kind and willing to go to treatment and I have had others who will scream, demand pain medication and be rude to staff. I don't see race play into it however that is from an ED standpoint and I think we have great peer recovery coaches who treat them all equal from what I can see.* -Participant 9  *I work in an ER where the patients are predominantly Black/African American and in my experience these patients have been offered treatment for OUD equally to patients of other races and are in concordance with best practices.-* Participant 12 |
| **Have you ever witnessed racial prejudice in job promotions/positions at your facility? (n=7)** | |
| No, I have not witnessed this or I can’t speak to a specific example | *The ER where I work has employees of many different races and backgrounds and in my experience, promotions have been given to those who deserve it unrelated to their race/ethnicity. There are leadership roles throughout our department of people of different race/ethnicity.* -Participant 12  *As I am not in a leadership position, I am not sure who has applied for jobs and who hasn't.* -Participant 23 |
| Yes, this is reflected in employee demographics | *Our entire C-suite are white men*. -Participant 4  *I work in an emergency department that has very few attending physicians of color. Most are white men. Most of the leadership in our group is white men.* -Participant 2 |
| **Have you ever witnessed Black/African American opioid use disorder patients being talked to in a disrespectful way?** | |
| Yes, especially among Black patients | *Asking patients how much they used in an unnecessary way--ie, "how much does that cost per day?" Irritation at withdrawing, vomiting patients when they are creating disruption in the clinical environment. –*Participant 8  *I have, but in general in the ED there is a very negative view of all patients with opioid use disorder and lots of negative things seem to be said regardless of race. Though I feel like there is a higher propensity for staff to say negative things about non-white patients*. -Participant 2  *I have witnessed aggression/disrespect towards “frequent flyers” by clinical staff familiar with patients* – Participant 25 |
| Patients with OUD are generally disrespected | *I'm not sure if it's any different than other races, but in general disparaged and disbelieved*. –Participant 24  *Yes, I’ve witnessed Black patients with OUD being talked to in a disrespectful way, most of our patients are black, amongst our patient population I don’t think that white people with OUD receive more or less disrespectful behavior*. -Participant 26 |
| **Have you ever witnessed Black/African American patients having opioid use disorder symptoms ignored by other doctors (i.e., pain, withdrawal, etc.)? (n=6)** | |
| Yes, though this happens to most patients regardless of race, and may be due to lack of training. | Again, I think this happens with all patients with opioid use disorder but probably happens more often in non-white patients.-Participant 2  Yes, I have seen providers ignore pain in patients with OUD assuming they are 'drug seeking'. – Participant 23  *Yes, I have witnessed this and I hope that this behavior is due to a lack of familiarity with how to treat withdrawal and being uncomfortable with treating OUD. However, I do understand that unconscious bias is real and it may be a double whammy to black patients, especially those with OUD. -*Participant 6 |
| No – have not witnessed | *This is not something I have personally witnessed in my department. We try to manage patient's pain (both with history of OUD and those with no history of OUD) to treat pain the best way possible while trying to avoid opioids for pain control unless absolutely necessary. We try very hard to help manage symptoms of withdrawal to keep patients comfortable and safe while treating other medical complaints and/or while assisting them with a more long term treatment solution for their OUD*. -Participant 10 |

**Appendix Table 4. Summary of major themes and subthemes from post-training survey, New Detroit Just Care Training participants (n=25), 2022**

| **Theme** | **Illustrative quote** |
| --- | --- |
| **Was this session useful to help you to communicate your own perceptions about race? (n=16)** | |
| Yes, but there wasn’t enough information about OUD | *Definitely useful regarding race disparities in healthcare and perceptions/mistrust of marginalized communities towards research. very little content specific to* OUD *however.* -Participant 8 |
| Yes, open dialogue was helpful in working through my own perceptions | *I felt that the open dialogue felt like a safe place to discuss individual perceptions regarding race and the conversation was very helpful in pointing things out that I may not have otherwise considered or thought were a big deal.* -Participant 21 |
| Yes, the historical context in medicine and systemic racism gave insight into mistrust, unconscious bias | *I was aware of some of the discrepancies with race and access to treatment resources and also to the manner in which non-white patients are treated. This course certainly furthered my understanding of these issues and deepened my understanding of other issues including African Americans’ mistrust of the government and the medical field as a whole*.-Participant 2  *There was a lot of helpful discussion during this session about racial disparities in healthcare. I found the history of medical mistrust within the Black/African American community to be very helpful at gaining a new perspective which in turn will help me be a better provider and advocate for my Black/African American patients better in the future.* -Participant 12 |
| **Was this session useful to help you to understand someone else’s perceptions about race? (n=16)** | |
| Yes, especially relevant to my own experience as a provider, other providers’, patient’s perspectives | *Yes!!!!! I think sometimes we get caught up in the busy day to day when working with patients in the ER we don't give them the time and consideration they deserve because we are so busy - this session really shed light on how little understanding I have when interacting with patients of other races - I plan on incorporating the perceptions learned today into my everyday practice.* -Participant 21  *It was important to see how other providers with privilege experience working with minority populations*. -Participant 15  Yes, I think the dialogue between everyone else involved in this group session and hearing their experiences with patients of other races was extremely helpful. -Participant 12 |
| Yes, in the context of systemic racism and historic events | I was introduced to several historical examples which helped me understand some of the factors leading to mistrust of the medical community from the perspective of African Americans. -Participant 2  Understanding my patient's perceptions of the house of medicine and government in general as not necessarily a trust endearing entity given recent history.-Participant 6 |
| **What new concepts and knowledge did you obtain from this session? (n=23)** | |
| Historical events that lead to mistrust of medical providers and persistent systemic racism | As someone actively involved in research and opioid use disorder, I think it is reasonable to bring up distrust of research and medicine with my patients in an attempt to give full disclosure and demonstrate that I understand why their may be mistrust in both research and medicine. -Participant 6  *I gained a lot of knowledge about the history of Blacks/African Americans and how they were utilized in experiments in the past and how they were mistreated in the process of these experiments. It helped gain a new perspective on the medical mistrust in Black/African American communities which in turn will help me be a better provider and advocate for them in the future.* -Participant 12  *I* *have a better understanding why a person from the African American community has a distrust of the medical community and may be less likely to accept my recommendations, especially being a white male. I hope in the future I will spend the extra time either asking questions or listening.-*Participant 19 |
| Disparities in incarceration | *Learned more about how a disproportionate number of black individuals are incarcerated for drug offenses than white individuals*. -Participant 23 |
| New language | *More familiarity with language around race and racism. Gained historical knowledge that created and enabled racist constructs*. -Participant 11 |
| **Did the training provide timely information or new insights relevant to your work? Why or why not? (n=23)** | |
| Yes, but need more insight or application to practicing medicine, treating OUD | *Helpful in recognizing unconscious bias and challenges facing patients of color who seek healthcare. Would have benefitted from more of a focus on OUD.-*Participant 11  *I really wish there was more of a focus on opiate use disorder in the Black community. There was almost none aside from questions raised by the group. There was discussion of substance abuse with cocaine/crack and war on drugs, but to extrapolate that into opiate use disorder in this patient population was just not done. Especially with current trends and bringing to center stage any health disparities in this patient population that we can work on addressing as a medical community.* -Participant 13 |
| Yes, historical events leading to medical mistrust was impactful | *Absolutely. It showed me that I likely have biases that I wasn't really aware of. While I may not be perfect overcoming them, I am going to take a step back with patients with OUD of color, and ask if I am treating them the appropriately*. -Participant 19  *I think that insights were not necessarily new but were a much needed reminder of the history behind treatment of Black individuals by the medical community- It was very relevant to my work - sometimes for example we get frustrated by individuals coming to the ER with what we perceive as not emergency issues- but the fact is they do not have the same access to care as we have, and the ER provider is the main source for medical care.* -Participant 21 |
| **Did the training provide timely information or new insights relevant to your personal life? Why or why not? (n=23)** | |
| Yes | *I think I could certainly take some of this information into my personal life. I feel like these inequities extend beyond the medical systems and now I can try to apply some of these concepts to broader issues.* -Participant 2  *Yes - in the group break out- I brought up that this session really made me want to get involved in community outreach to learn from [the] community we serve and have a more open dialogue regarding their thoughts. Although I answered that I thought I was comfortable talking to my patients about race- I probably am not - and it would sooooooo helpful to actually talk with them and actually hear them!* -Participant 21  *Yes, I think there is a constant struggle to understand the context of the disparities in health outcomes we see in the ED every day. [It] raised my awareness and empathy towards the lived experiences of my patients from disadvantaged populations.* -Participant 8 |
| No, more relevant to work than my personal life | *No, the training pertained more toward my work than personal life.* -Participant 23 |
| **Which part of the presentation was most useful? (n=23)** | |
| Small discussions | *Having the opportunity to discuss race with a small group in a safe space was very helpful for me to gain new perspectives and feel generally more comfortable with that type of discussion.* -Participant 12  *I think the later part, when we had a broad discussion about race inequalities. Listening to how the African American community feels toward the medical community and society in general was pretty eye opening.* -Participant 19 |
| Historical background | *I liked learning about the topics when we broke out into groups and delved into the history such as medical care access dating back to 1800 - to is unbelievable how black individuals were treated, used for experience and dehumanized. It is really going to make me change my thought and the day I approach individuals in my practice and in my everyday life!* -Participant 21  I thought the review of the problematic practices of research and the war on drugs were particularly informative. -Participant 8 |
| Videos and presentations of individual life experiences | *I thought the videos were well done as well as Jannah's sharing of her life experiences and lens*.-Participant 6 |
| **What topics do you think would be useful to explore in a future session? (n=24)** | |
| How opioid use disorder could be treated more effectively | *As I mentioned at the end of the session I personally would like the New Detroit perspectives on how to mobilize lessons learned in understanding unconscious bias and how to employ those in black patients with OUD.* -Participant 6  *As mentioned, very little time was dedicated directly to discussing treating opioid use disorder. It felt like part 1 of a 2 part discussion on care of patients with opioid use disorder.* -Participant 14  *I think spending more time specifically on racial disparities in receiving treatment for OUD as well as other common disease/chronic illness would be helpful as a health care provider.* –Participant 12 |
| Existing initiatives | *Learning more about specific and current initiatives that are ongoing to promote the equitable treatment of opiate use disorder in minority patients.* -Participant 25 |

**Appendix Table 5. Summary of major themes and subthemes from 2-month post-training survey, New Detroit Just Care Training participants (n=25), 2022**

| **Theme** | **Illustrative quote** |
| --- | --- |
| **In the past 2 months, have you witnessed treatment of Black/African American patients getting opioid use disorder treatment/treatment not in line with best practices? (n=16)** | |
| Yes, for various reasons | *It is common for " Frequent Flyer" patients who come to the ER to be treated quickly and with less empathy. Since the demographics of our patient population is urban AA patients, some get less than best practices. -Participant 1*  *I've seen patients left to wait longer than other patients. -Participant 18*  *Withholding pain medications because they are labeled as drug seeking. -Participant 4*  *Yelling at/fighting with patients out of frustration (especially in the setting of stupidly long wait times). -Participant 24*  *We rely on out "peer recovery" coaches to assist in arranging treatment. When they are not present, care seems to be less organized. – Participant 16*  *Have witnessed my black patients with OUD treated without kindness and with racial assumptions*. –Participant 5  *I think this was likely a lack of knowledge associated with OUD treatment and or OUD stigma. We’re not there yet knowledge wise nor in recognition of stigma.* – Participant 6 |
| No, good treatment | *I believe our hospital does a good job of addressing OUD regardless of race*. -Participant 7  *We have a largely black patient population with OUD and we have active programs to address OUD with a strong peer recovery team, research team to help with bupe dosing and a lot of support with discharge rescue kits with Narcan.* -Participant 13 |
| No, OUD patients are seen by someone else first | *As far as I know, all patients flagged for possible SUD are flagged and seen by our peer recovery team. -*Participant 3  *I have not witnessed this although we do not work in areas with another attending. –* Participant 23 |
| **In the past 2 months, have you witnessed Black/African American opioid use disorder patients being talked to in a disrespectful way? (n=14)** | |
| Yes | *I have seen some physicians speak to patients of varying races who have opioid use disorder in an inappropriate manner. I have not witnessed the use of any racial language recently, however*. -Participant 2  *I have seen these patients spoken to in a curt manner*. -Participant 18  *Our urban setting holds a demographic patient load of low income, less adequately insured patients who some staff speak poorly and disrespectfully as they find them aggressive and defensive.* –Participant 1  *Usually the conversations are shorter and questions asked are answered without kindness and in a rushed manner*.-Participant 5 |
| No | *Our providers treat all of our patients with the same level of respect regardless of race/ethnicity*. -Participant 12  *The majority of our ED patients are [B]lack, and we have programs to seek out these patients and try and acutely treat their OUD and condition. I have not witnessed anyone being rude.* -Participant 13 |
| **In the past 2 months, have you witnessed Black/African American patients having opioid use disorder symptoms ignored by other doctors (i.e., pain, withdrawal, etc.)? (n=16)** | |
| Yes, for various reasons | *… I think this was likely a lack of knowledge associated with OUD treatment and or OUD stigma. We’re not there yet knowledge wise nor in recognition of stigma*. -Participant 6  *Sickle cell patients are often ignored regarding their pain, and the majority of sickle cell patients are African American. I try to treat them aggressively with appropriate pain management, but sometimes the patients who return multiple days in a row can be difficult and make me think there is drug seeking behavior*. -Participant 19 |
| No | *I have not specifically witnessed this, although I believe it is likely under-recognized and not fully addressed unless prompted by the patient or our peer recovery specialists.* -Participant 5 |
| **Did attending the training impact your awareness of equitable treatment of Black/African American patients with opioid use disorder in your workplace? (n=21)** | |
| Yes, in various ways | *I think there’s definitely more empathy regarding a patient’s situation and more effort on trying to develop a plan of care when OUD is involved.-*Participant 3  *The training definitely opened my eyes to some of the disparities in care given to Black/African American patients. It also informed me of some of the reasons why Black/African American patients may less trusting of the care they receive and health care in general (and I can certainly understand why given some of the historical cases presented in the lectures).-*Participant 2  *Yeah, it made me realize that while I think I am treating all patients similar, there is a distrust of the medical community among the African American population which is understandable. I have been trying to spend a few extra minutes developing a relationship and letting them know they are being heard and trying to manage expectations from the beginning.* -Participant 19 |
| Yes but not for OUD | *This was more about historical racism towards [B]lack patients. There was minimal discussion about OUD. It was mainly about crack and systemic racism. I treated our patients with respect before, and if anything after this course it did bring to light the likely mistrust of myself as their provider.* -Participant 13  *I felt like there was little/no discussion around OUD in relation to race. Discussion was more focused on racial inequalities in general, but almost no content addressed OUD tx and race together.* -Participant 8 |
| **Did attending the training impact how you interacted with Black patients with opioid use disorder in the past 2 months? (n=20)** | |
| Yes | *I allow more time to discuss and consider barriers to interface with medical system and try to empathize with patients, specifically around factors influencing their OUD*. -Participant 11  *I certainly have thought about the training over the past couple of months, particularly when treating non-white patients. I would say I am more aware of my biases, however, I don't think my care has been altered in any significant way and I always go out of my way to treat my patients the same, to the best of my abilities.* -Participant 2  *I felt like I walked away from a few encounters feeling like patients hated the medical community more than previously remember being aware of.* -Participant 13 |
| **What** **specific needs are there to better serve Black patients with opioid use disorder in your ED**? **(n=25)** | |
| Better community resources, bias training for providers, education about historical background peer counselors representative of patient population | *Education is a big one. Understanding how Black/African American patients have been mistreated in the past (and present) will go a long way to provide equitable care to all of our patients. I think having open discussions with co-workers could also help to call attention to our biases and prevent any mistreatment of our patients.* -Participant 2  *I think that our ED does a very good job of treating Black patients with OUD. I think one of the biggest limitations is access to care after they leave the ED and programs in place for follow up and continued care would be the most beneficial. -*Participant 12 |
| **What specific needs are there to improve racial equity in health services in your community? (n=25)** | |
| Education, more equitable treatment access, outreach services, representation, addressing social determinants of health | *I think representation is something that is important. I think having non-white members of our teams is important, especially in leadership positions. Our work force should be representative of the populations that we take care of, and the population in which I work is very racially diverse.* -Participant 2  I think more education would help. Actually going into the community and giving basic medical education lectures at a community center. Informing people of what the actual capabilities of modern medicine are will help manage expectations and show them what standard of care is. -Participant 19 |
| **What stuck with you the most from the training? (n=23)** | |
| Historical information, harms done by medical community that inform medical mistrust, reflection on internal biases | *That drug addiction is a real disease and it really made me change the way I look at these individuals -right before I took this course I also watched the series Dopesick- there tends to be a lot of frustration with this particular group of people especially with drug-seeking behavior- and it is important to address that issue and just not stop medication.* -Participant 21  *Each encounter we have an opportunity to improve the relationship of black patients and some of their deep mistrust in medicine.* -Participant 7  *The historical factors contributing to a lot of the mistrust that Black/African American patients have of the health care system, and of the government as a whole.* – Participant 2 |
| **Michigan is now requiring racial equity training for emergency department practitioners. Do you think other states should require this training for their emergency department practitioners? Please explain your choice in the space provided below. (n=19)** | |
| Yes, but it should be outside Michigan too and include more about OUD | *Yes—[racial bias is an] important awareness to have and getting people more comfortable discussing is first step toward addressing inequalities in the health care system.* – Participant 8  *Yes I think it should but it should have more direct education to treatment of OUD in addition to the historical information shared. -*Participant 6  *This issue is not isolated to Michigan but is prevalent in many other states. Much of the inequity stems from miseducation and lack of exposure. This can be combated with training.-*Participant 4 |

**Appendix Table 6. Indicated Follow-up Actions by Participants - 2-Month Follow-Up Survey**

| **Question** | **Responses** | **N** |
| --- | --- | --- |
| Based on my experience with this conversation on race, I am considering the following actions (Check all that apply) | Engage in future conversations with either family, friends, or co-workers 22 | 22 |
|  | Apply this experience to engage the broader community in conversation on race 20 | 20 |
|  | Seek further outlets to learn more about how to build better race relations 23 | 30 |
|  | Too soon to apply this experience in a practical way 4 | 4 |
|  | Integrate this into my treatment practices with patients 22 | 22 |

**Appendix Figure 1. The Usefulness of Training in Communicating Participant’s Own Perceptions of Race –Post-Training Survey**

**A. Was this session useful to help you to communicate your own perceptions about race?**

**
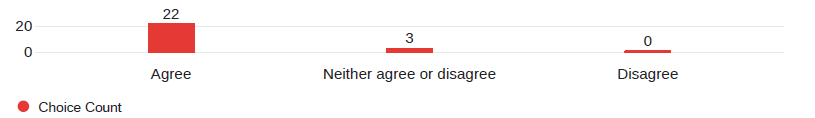
**

**B. Was this session useful to help you to understand someone else’s perceptions about race?**


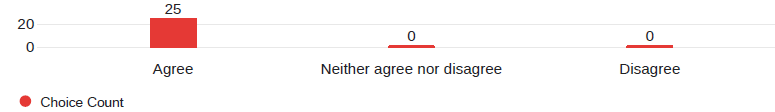


**Appendix Figure 2. Training Environment, Comfort, Racial Healing, and Awareness Impact Perceptions by Training Participants – Post Training Survey Responses**

1. **This no-fault environment was useful to reflect about the impact of race**

**
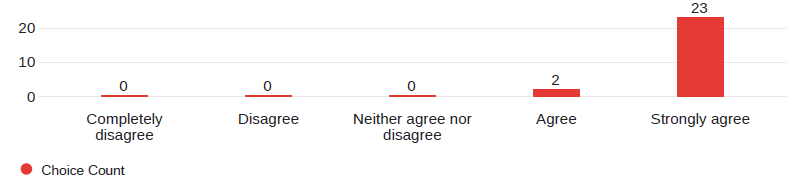
**

1. **I felt that a safe space was created to have this conversation on race**

**
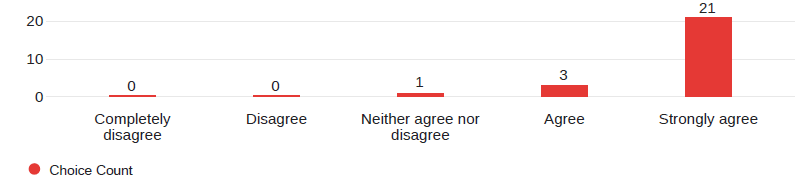
**

1. **This session increased my level of comfort to have a conversation about race using this type of small group format**

**
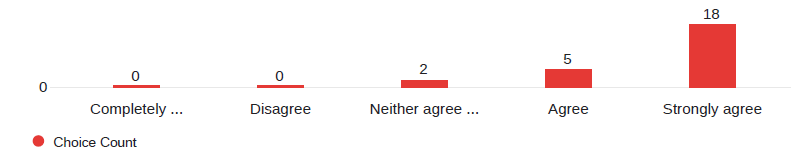
**

1. **This group dialogue today helped to promote racial healing**

**
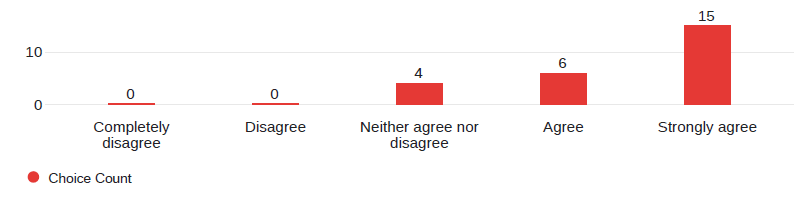
**

1. **This session increased my awareness of implicit or unconscious bias**


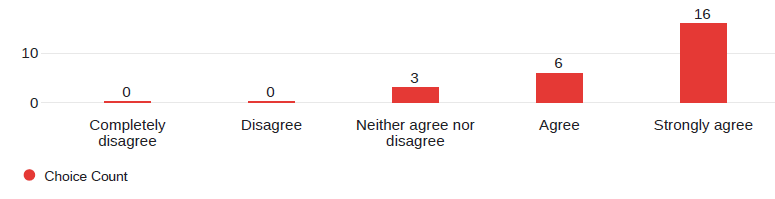

Supplement: qxae049_Supplementary_Data [file qxae049_supplementary_data.zip › Supplementary Appendix.docx]
